# Metronidazole-induced hepatotoxicity in a patient with xeroderma pigmentosum: A case report

**DOI:** 10.1097/MD.0000000000029416

**Published:** 2022-05-27

**Authors:** Jennifer Vanoli, Miriam Nava, Chiara Invernizzi, Fabio Panizzuti, Guido Grassi

**Affiliations:** a University Milano-Bicocca, Milan, Italy; b Clinica Medica, Department of Medicine and Surgery, University of Milano-Bicocca, Milan, Italy.

**Keywords:** acute hepatitis, antibiotics, case report, drug-induced liver injury, liver failure, xeroderma pigmentosum

## Abstract

**Rationale:**

Whereas metronidazole-induced hepatotoxicity is quite rare in the general population, in individuals carrying a nucleotide excision repair disorder, namely Cockayne syndrome, there is a high risk of developing this complication.

**Patient concerns:**

We report the case of a 44-year-old man, affected by xeroderma pigmentosum, who was admitted to the hospital presenting aspiration pneumoniae caused by worsening dysphagia and with severe hepatotoxicity during the hospitalization.

**Diagnoses:**

Acute hepatitis, which was leading to acute liver failure, occurred during antibiotic treatment with metronidazole and ceftazidime with an elevation of liver enzymes consistent with hepatocellular damage pattern.

**Interventions:**

Hydration with glucose 5% solution, pantoprazole and vitamin K were administered, meanwhile other causes of hepatitis were ruled out and the ongoing antibiotic treatment was stopped suspecting a drug-induced liver injury.

**Outcomes:**

Liver function nearly completely recovered 1 month later with a first rapid improvement, within few days, of aminotransferases and coagulation studies, and slower of cholestatic enzymes.

**Lessons:**

We describe the first case available in the literature of hepatotoxicity associated with metronidazole treatment in a xeroderma pigmentosum patient. Clinicians therefore, based on this report and according to the possible underlying mechanism shared by other genetic diseases characterized by alterations in the pathway of DNA-repair, should consider such adverse event also in patients affected by this rare disease.

## 1 Introduction

Xeroderma pigmentosum (XP) is a rare genetic autosomal recessive syndrome, characterized by an enzymatic defect in the DNA-repair pathway known as nucleotide excision repair (NER).^[1]^ NER is involved in the removal of bulky adducts induced by the ultraviolet component of sun-light or other environmental carcinogens. Alteration in this pathway results in several rare diseases characterized by a wide spectrum of clinical features with photosensitivity as a common trait. NER disorders include XP, trichothiodystrophy, Cockayne syndrome (CS), and cerebro-oculo-facio-skeletal-syndrome.^[2]^ Metronidazole (MTZ) is a nitroimidazole antibiotic which is widely used for gastrointestinal infections and dental complications. While MTZ-induced hepatotoxicity is quite rare in the general population, and MTZ is not listed among causes of drug-induced liver injury and acute liver failure, in individuals carrying a NER disorder, namely CS, there is a high risk of developing MTZ hepatotoxicity.^[3–6]^

Here we report the first case of a XP patient who developed severe hepatoxicity during the treatment with MTZ.

## 2 Case report

**Figure 1:**
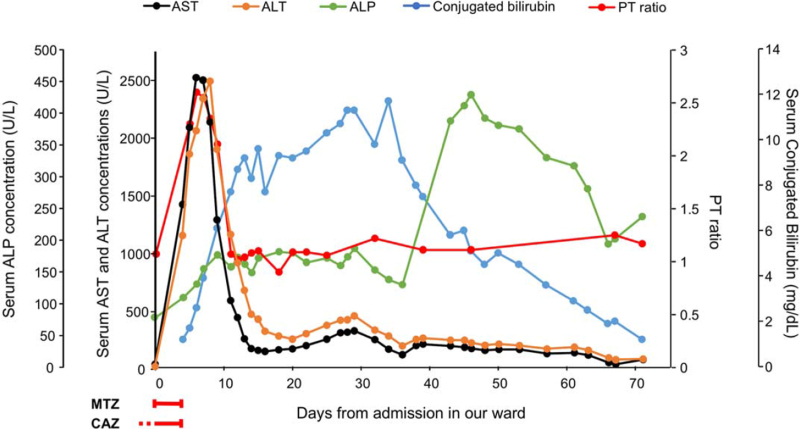
Evolution of serum concentrations of aminotransferases, cholestatic enzyme, and PT ratio after metronidazole-ceftazidime treatment from the admission in our ward. ALP = alkaline phosphatase, ALT = alanine aminotransferase, AST = aspartate aminotransferase, CAZ = ceftazidime, MTZ = metronidazole, PT = prothrombin.

A 44-year-old man, known to have XP with multisystemic involvement (cutaneous, ocular and neurological) diagnosed since the age of 2 years, was admitted to our medical department because of suspected aspiration pneumonia. The neurological involvement indeed, with a sensory and motor polyneuropathy, mixed cerebellar and sensory ataxia, hypoacusia and trigeminal neuralgia, led progressively to loss of autonomy in daily activities and dysphagia. This symptom worsened in the last few months before the admission, and treatment with escitalopram and alprazolam was started to control anxiety and panic attacks before meals. At the admission in our ward, an empiric antibiotic therapy with ceftazidime (CAZ) 2 grams every 8 hours was ongoing, started since the Emergency Room admission 2 days before because of acute respiratory failure and elevation of inflammation indices after inhalation at home. On presentation, the patient was alert, afebrile (axillary temperature of 37 °C), tachycardic (heart rate of 120 per min) with blood pressure of 160/100 mm Hg, SaO_2_ was 99% in high-dose oxygen from a reservoir mask at 15 L/min. A physical examination revealed bilateral reduced vesicular murmurs and neurological signs compatible with the underlying disease. Coagulation, renal and liver exams were normal. Computed tomography scan showed large pneumoniae involvement in left inferior lobe. Since other episodes of inhalation occurred, antibiotic therapy was potentiated with the introduction of MTZ 500 mg every 8 hours. Three days after the start of MTZ, severe abnormalities in liver function appeared with progressive elevation of liver enzymes and a prothrombin ratio greater than 1.5. As reported in Figure 1 both transaminases, aspartate aminotransferase and alanine aminotransferase (ALT), and cholestatic enzymes, conjugated bilirubin and alkaline phosphatase (ALP), were increased reaching an estimated R factor [ALT/upper limit normal ALT)/(ALP/upper limit normal ALP)] of 46, consistent with hepatocellular damage pattern (R factor > 5). Physical examination didn’t change from the admission except for jaundice. Investigation for viral markers (hepatitis A virus, hepatitis B virus, hepatitis C virus, cytomegalovirus, and Epstein-Barr virus) and for autoimmune hepatitis was negative. Hepatic ultrasound did not show abnormalities. Based on the clinical diagnosis of acute hepatitis which was leading to acute liver failure, hydration with glucose 5% solution, pantoprazole and vitamin K were started, nutrition was administrated through percutaneous endoscopic gastrostomy and antibiotic treatment was stopped suspecting a drug-induced liver injury (DILI) according to European Association for the Study of Liver Disease Guidelines. Aminotransferases reached their peak (2493 and 2140 U/L for ALT and aspartate aminotransferase, respectively) 5 days later and then started to decrease progressively, prothrombin ratio rapidly normalized, while cholestatic enzymes continued to increase for the next 3 weeks reaching the value of 11 to 12 mg/dl for conjugated bilirubin. An abdominal magnetic resonance imaging excluded obstructive jaundice. We did not perform a liver biopsy because of deteriorated general condition, however, without specific therapy, cholestatic enzymes started to decrease gradually and liver function nearly completely recovered one month later. Unfortunately, the patient died due to severe complications of nosocomial pneumonia. The patient provided an informed consent for publication of data, including in this case report. An approval by ethics committee was not necessary because of the routine health care.

## 3 Discussion

According to Roussel Uclaf Causality Assessment Method in assessing causality of drug induced liver injury,^[7]^ the acute liver damage observed in our XP patient may be ascribed to the treatment with MTZ and CAZ. The time to onset reaction and the improvement after cessation of the treatment were highly suspicious of this association, and other causes of acute hepatitis were ruled out. Both MTZ and CAZ can be equally suspected, but data available in the literature suggest the predominant role of MTZ. The cephalosporins, indeed, have been associated with little hepatotoxicity and only rarely DILI due to these agents has been published in general population,^[6]^ mostly confounded by usage of other potential hepatotoxic medications^[8]^ and with no other cases reported selectively in patient affected by NER disorders. A special exception is ceftriaxone which has a unique clinical phenotype of liver injury with biliary sludge and stones due to crystallization of ceftriaxone in the biliary tract that we did not observe in our case. Whereas acute and clinically relevant liver injury from MTZ is also rare and MTZ is not listed among causes of DILI and acute liver failure, this has been reported in case of individuals with CS.^[3–6]^ CS is a NER disorder characterized by profound postnatal decline of somatic and brain growth, with characteristic *facies* (sunken eyes and cheeks, decreased tearing and corneal scarring, miotic pupils, a sharp nose, a jutting chin, thin lips, inadequate salivation and dental caries) and a poor prognosis (patients with CS have a very short life expectancy and rarely survive > 30 years).^[9]^ As previously mentioned, there are clinical reports of MTZ hepatotoxicity in CS. These cases displayed a short latency (1–7 days) to onset of jaundice, a hepatocellular pattern of enzyme elevations and a severe course with a high mortality rate. The mechanisms of MTZ hepatotoxicity are not known. MTZ is a prodrug metabolized in the liver by oxidative processes through the reduction of its nitrogroup and cyto-toxicity may be activated by deleterious interactions with DNA resulting in DNA strand breaks in susceptible microorganisms.^[10]^ The pathogenic mechanism favouring MTZ hepatoxicity in CS patients is undetermined but the constitutional DNA repair disorder affecting these patients may play a role, considering also that an underlying hepatic dysfunction is not unusual in CS patients.^[11]^ The association between CS and MTZ hepatotoxicity is now well-known and reported as an alert in the MTZ datasheet by various drug regulatory agency such as Emergency Medicines Agency and Food and Drug Administration.

In our case, the first phase of the liver damage presented with a pattern of hepatotoxicity like the one typical of CS: short latency (the first laboratory abnormalities were detected after 3–4 days), a hepatocellular pattern (*R* value >5) and a severe course (high risk of liver failure). We supposed that the underlying mechanism of injury might be similar and related to the defect in the pathway of DNA-repair since both CS and XP belong to the group of NER disease. In the second phase, the prolonged cholestatic damage pattern that we observed, without dilation of the biliary tract and despite the gradually parallel decrease of aminotransferases, remains unclear. We considered the hypothesis of a vanishing bile duct syndrome secondary to DILI, which self-resolved, but could not be confirmed through a liver biopsy and we acknowledge this is the limit of the present case report.

In summary, we presented the first case available in the literature of hepatotoxicity associated with MTZ treatment in a XP patient. Clinicians therefore, based on this report and according to the possible underlying mechanism shared with other genetic diseases characterized by alterations in the pathway of DNA-repair, should consider such adverse event also in patients affected by this rare disease.

## Author contributions

**Conceptualization:** Chiara Invernizzi, Jennifer Vanoli, Miriam Nava.

**Data curation:** Chiara Invernizzi, Fabio Panizzuti, Miriam Nava.

**Supervision:** Guido Grassi, Jennifer Vanoli.

**Validation:** Guido Grassi.

**Writing – original draft:** Miriam Nava.

**Writing – review & editing:** Guido Grassi, Jennifer Vanoli.
